# Job burnout on subjective wellbeing among clinicians in China: the mediating role of mental health

**DOI:** 10.3389/fpsyg.2023.1227670

**Published:** 2023-09-15

**Authors:** Yingjie Fu, Derong Huang, Shuo Zhang, Jian Wang

**Affiliations:** ^1^Centre for Health Management and Policy Research, School of Public Health, Cheeloo College of Medicine, Shandong University, Jinan, Shandong, China; ^2^NHC Key Lab of Health Economics and Policy Research, Shandong University, Jinan, Shandong, China

**Keywords:** subjective wellbeing, job burnout, mental health, clinicians, mediating effect

## Abstract

**Background:**

Although job burnout and mental health difficulties are prevalent negative influences on clinicians’ subjective wellbeing (SWB), there are few investigations into their relationships. This research investigates the mediating role of mental illness in the association between clinicians’ SWB and job burnout in China.

**Methods:**

This study used the data collected from a cross-sectional survey conducted in China. Using convenience sampling, we conducted a face-to-face questionnaire survey among clinicians in a tertiary hospital in Shandong Province from August to September 2019. The 22-item Maslach Burnout Inventory-Human Service Survey (Chinese version) and the Personal Wellbeing Index-Adult assessed job burnout and SWB. The Chinese short version of Depression, Anxiety and Stress Scale (DASS-C21) assessed mental health. We also collected data on participants’ sociodemographic characteristics and job-related factors. Structural equation modeling (SEM) was applied to examine the associations between variables.

**Results:**

Among the 422 participants, 80.8% of the participants reported at least one symptom of job burnout, whereas 5.7% reported all three symptoms of burnout. The prevalence rates of depression, anxiety, and stress were 40.3, 41.7, and 24.9%, respectively. Only 12.8% of the participants had high level of SWB. In mediation analysis, job burnout is positively associated with mental illness (β = 0.809, *P* < 0.001), mental illness had a significant negative association with SWB (β = −0.236, *P* = 0.013), and a negative association between job burnout and SWB was significant (β = −0.377, *P* = 0.002). Mental illness played a partially mediated role in the association between job burnout and SWB (indirect effect = −0.191, 95% CI: −0.361∼−0.017), and the mediating effect of mental illness can explain the 33.6% of the total effect of job burnout on SWB.

**Conclusion:**

This study provides evidence that the effect of job burnout on SWB is partially mediated by mental illness among clinicians in China. Medical administration departments and hospital administrators should pay close attention to the job burnout and mental health of clinicians, so as to effectively improve the SWB of clinicians.

## Introduction

As the main provider of medical and health services, the stability of the professional team of clinicians is an important prerequisite for ensuring the quality of medical services (Fang et al., 2014). Clinicians are also the “cornerstone” of the healthy development of medical and health services. In China, clinicians in tertiary public hospitals, as the most precious power resources of hospitals, are the concentrated expression of the core competitiveness of the development of hospitals. Tertiary public hospitals in China not only undertake the treatment of difficult and complicated diseases in their region, but also undertake the clinical education work of leading the medical development and training the next generation of excellent clinicians, whose importance to the development of regional health and education is evident (Thepapernews, 2015).

Since the introduction of positive psychology, the study of individual subjective wellbeing (SWB) has become increasingly popular, especially for individuals within specific organizations. SWB is a comprehensive evaluation of the individual’s emotional life quality and cognitive intelligence, reflecting the individual’s social function and adaptive state (Diener et al., 2018). Human resources are fundamental to the survival and sustainable growth of modern organizations (Li et al., 2022). Clinicians are an important human capital of hospitals, and their SWB is a practical issue that medical management departments and hospital managers must pay attention to. The SWB of clinicians is especially vital in the healthcare system, affecting their own physical and mental health and career development (Goitein, 2014; Nojima et al., 2015; Zhou et al., 2018; Koch et al., 2020), and ultimately, the quality of medical services and patient satisfaction (Friedberg et al., 2014; Scheepers et al., 2015; Wang et al., 2022). However, healthcare professionals commonly experience job burnout and mental health challenges, which can detrimentally affect their SWB.

Medical professionals face significant demanding in their practice, as they are required to promptly and accurately address the needs of patients and families. In addition, night work, shift work and long working hours are prevalent in the medical profession. Compared to the general working population, many clinicians often experience job burnout as they deal with high levels of job stress and emotional demands (Prins et al., 2007a,b; Wallace et al., 2009; Dyrbye et al., 2013). Many researches have demonstrated that chronic exposure to work-related stress can lead to job burnout (Schaufeli et al., 1993; Collings and Murray, 1996). Job burnout can be defined as a psychological syndrome, which is a long-term response to long-term interpersonal stress at work (Schaufeli and Greenglass, 2001). It was initially measured by Maslach and Jackson (1981) and defined as a state of emotional exhaustion (EE), depersonalization (DP), and low personal accomplishment (LPA). EE refers to reduced energy levels, extreme fatigue, exhaustion, energy loss, depletion and weakness (Maslach and Leiter, 2016). DP, also known as cynicism, involves adopting a negative or detached attitude toward clients, including irritability, loss of idealism, and withdrawal (Maslach and Leiter, 2016). LPA is represented by a decline in personal achievement, also known as inefficiency, which is described as reduced productivity or ability, low morale and a sense of inefficiency (Maslach and Leiter, 2016). The 11th edition of the International Classification of Diseases (ICD-11), introduced in 2019, officially recognized job burnout as a multidimensional syndrome encompassing emotional exhaustion, depersonalization, and diminished feelings of personal accomplishment (Xiao et al., 2022). Job burnout is common among healthcare professionals, especially those who provide healthcare on the front lines. Previous studies have shown that the prevalence rate of job burnout symptoms among Chinese doctors, ranging from 66.5 to 87.8% (Lo et al., 2018).

It is no secret that the association between job burnout and mental illness is close (Woon and Tiong, 2020). Mental illness is a complex public health issue with extensive social and economic implications and serious consequences for physical health (Stenius, 2007). The Chinese chronic disease cohort survey has shown that the mental health of Chinese adults is not optimistic, especially anxiety and depression symptoms (Chen et al., 2017). Many studies have shown a high prevalence of mental health problems, such as depression and anxiety in the health service. Xinhua et al. (2006) and Shen et al. (2012) found that 27.7% of clinicians had mental health problems and 37.1% of clinicians had depression in general hospitals in China. Yinuo (2020) used the 21-item Depression, Anxiety and Stress Scale (DASS-21) to screen clinicians in China and reported rates of 40.98% for depression, 45.77% for anxiety, and 25.88% for stress. The Malaysian study, which also used DASS-21, found that the prevalence of depression, anxiety and stress among medical staff was 18.7, 38.6, and 12.0%, respectively (Woon and Tiong, 2020). A national database study in Korean revealed that healthcare workers had higher odds ratios for mood and anxiety disorders than the employee in other workplaces (Kim et al., 2018). At the same time, job burnout has emerged as a significant factor affecting individual SWB. A systematic review of 19 studies found that job burnout may negatively impact on the wellbeing of human service workers, including healthcare providers (Williford et al., 2018). Previous research has indicated that among medical personnel, burnout has detrimental effect on health status and overall wellbeing, which can lead to depressive symptoms and suicidal ideation (West et al., 2009; Dyrbye and Shanafelt, 2011; Shanafelt et al., 2016; Williford et al., 2018). For example, mental illness was shown to associate with job burnout negatively and have a significant mediating effect in the association between job burnout and poor quality of life among medical staff in a Malaysian hospital (Woon and Tiong, 2020). A study found that job burnout and SWB exhibited significant negative correlation among female doctors in Chinese hospital (Wang et al., 2020). In addition, job burnout has been linked to job dissatisfaction, frequent turnover, and increased in medical malpractice or errors. Therefore, job burnout is a potential obstacle to the mental health and SWB of medical staff and health care quality.

**FIGURE 1:**
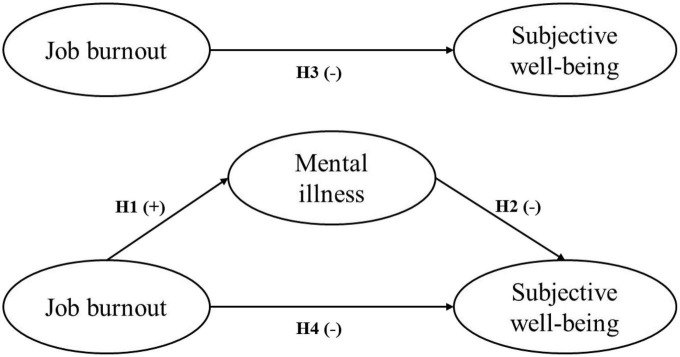
Theoretical model and hypotheses.

In the Chinese context, researches specifically focused on job burnout among clinicians, and the associated issues of mental health and SWB, is lacking. In conjunction with the previous arguments, we proposed the following research hypotheses: Hypothesis 1: Job burnout is positively related to mental illness. Hypothesis 2: Mental illness is negatively related to SWB. Hypothesis 3: Job burnout is negatively related to SWB. Hypothesis 4: Job burnout is negatively related to SWB through the mediation of mental illness. The purpose of this study was to explore how job burnout influenced SWB through the mediating roles of mental illness. Our proposed model is summarized in Figure 1. The finding will enrich relevant theory and provide a new perspective for hospital human resource management.

## Materials and methods

### Participants

Our study was conducted in a hospital in Shandong Province from August to September 2019. All investigators received professional training on the questionnaire survey a few days before the formal investigation. Skilled or trained teachers and graduate students from Shandong University personally conducted structured face-to-face interviews with the survey participants in the relevant clinical departments of the hospital, with the assistance of medical administration department workers. Participants were selected by using convenience sampling method. This survey did not include intern doctors, standardized training doctors, training doctors from other hospitals and those who worked in the sample hospital for less than 1 year. The Ethics Committee of the first author’s university approved this cross-sectional study (ECSHCMSDU20190303), and it adhered to the tenets of the Declaration of Helsinki. Informed consent was obtained from all participants prior to entry into this study. We obtained written informed consent from 431 clinicians. However, nine questionnaires were excluded due to its unavailability. Finally, 422 clinicians were included in this study.

### Measures

#### Sociodemographic and job-related variables

Sociodemographic variables included gender, age, marital status (married/cohabiting, and single including never married, divorced, and widowed), number of children and educational level. Job-related variables included working years, specialty, professional title, annual income, academic environment, work environment and welfare benefits.

#### PWI

Subjective wellbeing was explored using the Personal Wellbeing Index-Adult (PWI), which consists of seven items, with a response scale of 0 (completely unsatisfied) to 10 (completely satisfied) (International Wellbeing Group, 2006; Casas et al., 2008). The items refer to satisfaction with health, standard of living, life achievements, sense of security, the groups of people of which they part in, future security and relationships with others (Navarro Moya et al., 2017; Jovanovic et al., 2019). In this study, the seven items included health, standard of living, work achievements, safety, community involvement, future security, and personal relationships. The internal consistency analysis of the PWI for our sample indicates a Cronbach’s alpha of 0.924, which is consistent with values obtained in other studies ranging from 0.70 to 0.87 (Navarro Moya et al., 2017; Jovanovic et al., 2019).

#### MBI-HSS

The Chinese version of the Maslach Burnout Inventory-Human Service Survey (MBI-HSS) was utilized to measure job burnout in this study, which consists of 22 items, with each item rated on a scale of 0 (never) to 6 (daily) (Chaoping et al., 2003). The scale was divided into three dimensions of EE, DP and LPA. As there are different thresholds for job burnout, the high score in each domain was designated as follows: EE score ≥ 27, DP score ≥ 13, and LPA score ≤ 31 (Rotenstein et al., 2018). The internal consistency coefficients of the three sub-scales range from 0.80 to 0.89 (Maslach et al., 2001). Based on the previous studies, one or more exacerbated dimensions in the MBI-HSS are considered an indication of the presence of professional burnout (Maslach et al., 2001; Chaoping et al., 2003; Rotenstein et al., 2018). The internal consistency of this study was measured using Cronbach’s alpha, producing values of 0.937 for EE, 0.928 for DP, 0.902 for LPA, and 0.859 for the total MBI-HSS.

#### DASS-C21

Mental health status was measured by the Chinese Short Version of the Depression, Anxiety and Stress Scale (DASS-C21) (Taouk et al., 2001). The scale includes three sub-scales of depression, anxiety and stress, with a total of 21 items, which respectively measure individuals’ experience of negative emotions such as depression, anxiety and stress. The internal consistency coefficient of the three sub-scales is between 0.80 and 0.83 (Yi et al., 2012). The internal consistency coefficient of depression, anxiety and stress sub-scales in this study was 0.892, 0.882, and 0.876, respectively. A 4-level score from 0 to 3 was used (“0” was “ not consistent,” “1” was “partially consistent,” “2” was “mostly consistent,” and “3” was “always consistent”). The total score of each sub-scale multiplied by two was the score of the sub-scale, and the score ranged from 0 to 42. The higher the score was, the more serious the corresponding negative emotion was (Taouk et al., 2001). The cut-off points used for self-reported depressive, anxiety, and stress symptoms in their respective sub-scales were >13 for Depression, >9 for Anxiety, and >18 for Stress, which indicated at least moderate level of symptom severity (Taouk et al., 2001; Yi et al., 2012).

### Statistical analyses

Data analysis was performed using SPSS version 24.0 (IBM Corp., Armonk, NY, USA) and AMOS 23.0 (IBM Corp., Armonk, NY, USA). Frequencies, percentages, means and standard deviations were performed to describe the socio-demographic characteristics of clinicians, job burnout, mental health, and SWB. A one-way analysis of variance (ANOVA) or student’s *t*-test was used to compare the differences in the total SWB scores in each group, which are described by different demographic characteristic. Person correlation coefficients were calculated to examine the correlation between job burnout, mental health, and SWB. Finally, SEM was utilized to test the hypothesized interrelationships among the three key variables. The sub-scale scores of job burnout, mental health and SWB were used as indicators of each latent variable. The model was constantly refined to verify model fit and the best-suited model was selected. Indicators to evaluate model fit in the current study included the relative chi-square (χ^2^/df) statistic, the standardized root-mean-square residual (SRMR), the root mean-square error of approximation (RMSEA), the goodness-of-fit index (GFI), the comparative fit index (CFI), and the normal fit index (NFI). The evaluation of the model fit was based on the following criteria: χ^2^/df < 3, SRMR < 0.08, RMSEA < 0.05, GFI > 0.90, CFI > 0.90, and NFI > 0.90 (MacKinnon et al., 2002). Significance testing of each mediating pathway was also performed according to methods proposed by MacKinnon et al. (2002). Statistical significance was set at *P* < 0.05.

## Results

### Descriptive statistics of demographic characters

The proportion of female was slightly higher than male (50.9 vs. 49.1%). The mean age of clinicians was 36.28 ± 8.05. Most clinicians were married with spouses (83.9%), and more than half had raised at least one child (71.6%). The majority of clinicians had an education level above bachelor degree (89.1%), and have worked for more than 5 years (65.6%). More than half of the clinicians had an per capita annual income less than 160,000 Chinese yuan (79.7%). In addition, 30.1, 21.1, and 26.5% of clinicians were satisfied with the academic environment, work environment, and welfare benefits, respectively. Table 1 shows more detailed information on the socio-demographic characteristics and job-related characteristics of clinicians.

**TABLE 1:** Descriptive results of participants characteristic.

Characteristic	*N*	%	PWI	*t/F*	*P*-value
**Gender**					
Male	207	49.1	63.59 ± 15.66	0.452	0.652
Female	215	50.9	62.90 ± 15.80		
**Age (years)**					
26∼30	117	27.7	63.20 ± 13.36	4.335	0.005
31∼35	132	31.3	59.85 ± 16.65		
36∼40	72	17.1	63.95 ± 14.94		
41∼	101	23.9	67.20 ± 16.73		
**Marital status**					
Single	68	16.1	61.22 ± 15.11	−1.193	0.236
Married/cohabited	354	83.9	63.62 ± 15.82		
**Number of children**					
0	120	28.4	63.07 ± 14.56	2.670	0.070
1	226	53.6	64.51 ± 15.23		
2	76	18	59.72 ± 18.37		
**Educational level**					
Bachelor’s degree	46	10.9	60.00 ± 15.67	2.329	0.099
Master’s degree	246	58.3	62.71 ± 15.14		
Doctorate	130	30.8	65.37 ± 16.62		
**Working years**					
1∼5	145	34.4	63.61 ± 13.18	5.048	0.001
6∼10	142	33.6	59.36 ± 16.66		
11∼15	40	9.5	67.46 ± 14.91		
16∼20	31	7.3	62.53 ± 17.03		
21∼	64	15.2	68.71 ± 16.75		
**Specialty**					
Internal medicine	125	29.6	63.19 ± 15.35	1.843	0.047
Surgery	105	24.9	62.75 ± 14.67		
Obstetrics and gynecology	43	10.2	67.04 ± 13.61		
Pediatrics	37	8.8	60.66 ± 17.91		
Emergency medicine	23	5.5	59.88 ± 16.47		
Anesthesiology	18	4.3	55.08 ± 17.87		
Others	71	16.8	66.92 ± 16.48		
**Professional title**					
Primary	168	39.8	62.30 ± 13.80	9.089	<0.001
Intermediate	143	33.9	60.50 ± 17.09		
Senior	111	26.3	74.54 ± 13.78		
**Annual income (Chinese yuan)**					
60,000∼100,000	182	43.4	60.15 ± 15.00	11.177	<0.001
110,000∼150,000	154	36.3	62.54 ± 16.11		
160,000∼200,000	60	14.2	69.12 ± 14.83		
210,000∼	26	6.2	75.38 ± 10.62		
**Academic environment**					
unsatisfied	116	27.5	55.84 ± 19.18	14.073	<0.001
general	179	42.4	65.62 ± 13.07		
satisfied	127	30.1	66.64 ± 13.34		
**Work environment**					
unsatisfied	118	28	59.94 ± 18.50	3.771	0.025
general	215	50.9	63.92 ± 14.80		
satisfied	89	21.1	65.95 ± 13.07		
**Welfare benefits**					
unsatisfied	187	44.3	58.63 ± 16.90	16.824	<0.001
general	123	29.1	65.13 ± 13.48		
satisfied	112	26.5	68.84 ± 13.69		

### SWB among clinicians with different characteristics

Table 1 also displays the SWB among clinicians with different characteristics. The PWI scores of most of these groups were distributed between 55 and 75. Results revealed statistical differences in the other variables except for gender, marital status, number of children, and educational level. The participants who belonged to such characteristic group as: age at least 41 years old, having one child, working years at least 21 years, Obstetrics and gynecology, senior professional title, annual income at least 210,000 Chinese yuan, satisfied with the academic environment, satisfied with the work environment, satisfied with the welfare benefits, had higher PWI score.

### Job burnout, mental health, and SWB among clinicians

Table 2 presents the descriptive results of the study variables. The mean scores of the three dimensions of job burnout were 21.87 ± 1.00 (EE), 6.20 ± 1.07 (DP), and 22.16 ± 0.94 (LPA), respectively. Among the 422 participants, 24.2% experienced high level of EE, 10.2% experienced high level of DP, and 77.7% had a high sense of LPA. Overall, 80.8% of the participants reported at least one symptom of job burnout, whereas 5.7% reported all three symptoms of burnout. Mental illness included depression, anxiety and stress symptoms, with an average score of 8.79 ± 7.98 (Depression), 7.94 ± 7.90 (Anxiety), and 11.73 ± 8.00 (Stress), respectively. When considered these symptoms collectively, 198 respondents had experienced mental illness, indicating the presence of at least one of these symptoms, accounting for 46.9%. Among them, 170 respondents with various levels of depressive symptoms made up 40.3% of the group; 176 respondents with various levels of anxiety symptoms made up 41.7%; and 105 respondents with various levels of stress symptoms made up 24.9%. The mean score of the SWB of clinicians was 63.24 ± 15.72. Only 12.8% of the clinicians had high level of SWB. The proportions of clinicians who had low and moderate levels of SWB were 36.6 and 50.9%.

**TABLE 2:** Descriptive results of the job burnout, mental health, and SWB among clinicians.

Variables	M ± SD
Job burnout	50.21 ± 16.17
EE	21.87 ± 1.00
DP	6.20 ± 1.07
LPA	22.16 ± 0.94
**Mental health**	
Depression	8.79 ± 7.98
Anxiety	7.94 ± 7.90
Stress	11.73 ± 8.00
**SWB**	
Health	6.51 ± 1.99
Standard of living	6.27 ± 1.78
Work achievements	5.92 ± 1.89
Safety	6.36 ± 1.83
Community involvement	5.87 ± 2.07
Future security	6.37 ± 1.92
Personal relationships	6.95 ± 1.78
PWI total score	63.24 ± 15.72

M, means; SD, standard deviation; EE, emotional exhaustion; DP, depersonalization; LPA, low personal accomplishment; SWB, subjective wellbeing.

### Correlation analyses of job burnout, mental illness and SWB

The correlation coefficients are presented in Table 3. Job burnout, depression, anxiety and stress were positively correlated with each other (*P* < 0.01). Additionally, those factors and SWB had a negative correlation (*P* < 0.01).

**TABLE 3:** The descriptive analysis and correlations among relevant variables.

Variables	Job burnout	EE	DP	LPA	Depression	Anxiety	Stress	SWB
Burnout	1.000							
EE	0.820**	1.000						
DP	0.756**	0.577**	1.000					
LPA	0.629**	0.158**	0.225**	1.000				
Depression	0.635**	0.601**	0.554**	0.253**	1.000			
Anxiety	0.600**	0.567**	0.518**	0.242**	0.848**	1.000		
Stress	0.571**	0.584**	0.447**	0.212**	0.851**	0.83**	1.000	
PWI	−0.550**	−0.472**	−0.384**	−0.344**	−0.544**	−0.499**	−0.460**	1.000

**P < 0.01; EE, emotional exhaustion; DP, depersonalization; LPA, low personal accomplishment; SWB, subjective wellbeing.

### Mediation analyses

**FIGURE 2:**
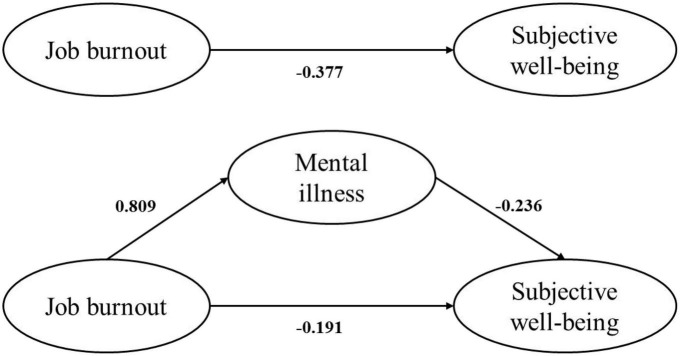
Structural equation analysis of job burnout, mental illness, and SWB.

Structural equation modeling was used to quantify the hypothesized interrelationships among the three study variables. The results of SEM and hypothesis testing are presented in Tables 4, 5 and Figure 2. Tables 4, 5 summarize the results of the direct, indirect, and total effects of the study variables. Standardized coefficients representing the direct associations between variables are displayed over the arrows in Figure 2. Regarding the quality of fit, the overall model fit indices of the valid model in Figure 2 suggested a satisfactory level with χ^2^/df = 2.379, SRMR = 0.000, RMSEA = 0.057, GFI = 0.984, CFI = 0.992, and NFI = 0.987.

**TABLE 4:** Structural model assessment and their corresponding results.

	Standardized regression coefficient	*P*-value	Result
Jon burnout→mental illness (H1)	0.809	<0.001	Support
Mental illness→Depression	0.939		
Mental illness→Anxiety	0.906	<0.001	
Mental illness→Stress	0.908	<0.001	
Job burnout→LPA	0.269		
Job burnout→DP	0.718	<0.001	
Job burnout→EE	0.796	<0.001	
Mental illness→SWB (H2)	−0.236	0.013	Support
Job burnout→SWB (H3)	−0.377	0.002	Support

EE, emotional exhaustion; DP, depersonalization; LPA, low personal accomplishment; SWB, subjective wellbeing.

**TABLE 5:** Direct, indirect, and total effects of study variables.

Model pathways	Standardized β coefficient	95% CI	
		Lower	Upper
**Total effects**			
Job burnout→mental illness	0.809	0.710	0.895
Mental illness→SWB	−0.236	−0.440	−0.029
Job burnout→SWB	−0.568	−0.673	−0.458
**Direct effects**			
Job burnout→mental illness	0.809	0.710	0.895
Mental illness→SWB	−0.236	−0.440	−0.029
Job burnout→SWB	−0.377	−0.649	−0.151
**Indirect effects**			
Job burnout→mental illness→SWB	−0.191	−0.361	−0.017

SWB, subjective wellbeing.

As expected, the results revealed a significant positive association between job burnout and mental illness (β = 0.809, *P* < 0.001), which meant H1 should be accepted. Mental illness had a significant negative association with SWB (β = −0.236, *P* = 0.013), thus supporting H2. The results also indicated that the direct negative association between job burnout and SWB was significant (β = −0.377, *P* = 0.002), thus supporting H3 (Table 4).

As shown in Table 5, the indirect association between job burnout and SWB was significant. The 95% confidence interval (95% CI) of mediating effects of the Job burnout→Mental illness→SWB pathway was (−0.361, −0.017), which did not include 0, indicating that mental illness mediated the indirect relationship between job burnout and SWB, the mediating effect of mental illness can explain 33.6% of the total effect of job burnout on SWB, thus supporting H4.

## Discussion

In this study, we investigated the prevalence of job burnout and mental health among clinicians in a tertiary hospital and their SWB. Consistent with our hypotheses, a positive correlation was found between job burnout and mental illness, while a negative correlation was observed between job burnout and SWB. Mediation analysis revealed that mental illness mediated the association between job burnout and poor SWB among clinicians. Notably, this study represents the first attempt to comprehensively investigate job burnout, mental health, and SWB among clinicians in a Chinese hospital, adding to the existing literature in this field.

Job burnout has been common among healthcare professionals, especially those providing health care at the front line. Studies have shown that regardless of specialties among clinicians, the rates of job burnout symptoms ranged from 36 to 51% in the Western country (Medscape, 2019). A nationwide survey on physician job burnout in China has shown that 31.28% of physicians experienced job burnout (Wu et al., 2021). Based on the findings in this study, the prevalence of participants experienced at least one job burnout symptom was 80.8%, which was higher than that reported in Western countries and Chinese nationwide survey. This finding was consistent with a study by Qianqian and Aiyun (2017), which reported the job burnout rate was 76.64% among clinicians in tertiary hospitals in Yunnan Province, China. The rates of different categories of job burnout identified among participants of this study were different from previous studies using the same assessment tool and cut-off points, especially on the LPA. Our results showed that 77.7% of participants had a high sense of LPA, which is much higher than in other studies in China. For example, studies in Yunnan and Fujian Province, China both found clinicians had low or moderate sense of LPA in tertiary hospitals (Guozhong, 2014; Qianqian and Aiyun, 2017). Another study in Jinan, China showed that 79.52% of clinicians had a high sense of LPA in 2014 (Xiaojuan and Fuzhong, 2015), which was close to our results. At the same time, Chinese oncologists have similar job burnout rates as their American counterparts, but a greater loss of LPA (Ma et al., 2019). These inconsistent findings may be explained by the individuals’ varied geographic location and work environments.

In this study, 46.9% of participants were screened positive for at least one of the mental illness symptoms: depression, anxiety, and stress. This result is very similar to the result of Malaysian hospital (41.8%) (Woon and Tiong, 2020). In terms of dimensions, the prevalence rates of self-reported depressive, anxiety, and stress symptoms were 40.3, 41.7, and 24.9%, respectively, which was consistent with a nationwide survey among medical staff in China. It can be seen that the mental health of medical staff is not optimistic, especially depression and anxiety. Foreign surveys have shown that 57–73% of clinicians have depression in the general sense, that is, depression, melancholy or sadness, with the highest proportion in Germany, Portugal and the United States, and the lowest proportion in Britain. French, Spanish and British clinicians had the highest rates of clinical depression (i.e., a state of severe depression that lasts for a period of time), while German clinicians had the lowest rates (Medscape, 2019). Despite the fact that these findings were not directly comparable, they are adequate to demonstrate that clinicians’ mental health issues need attention from the wider community.

The PWI used in this study was divided into seven aspects: health, standard of living, work achievements, safety, social participation, future security and personal relationships. As can be seen from the measurement results, the clinicians scored the highest in the personal relationships, indicating that the clinicians in the sample hospital were most satisfied with the current harmonious colleague relationship and good cooperation atmosphere. This is also related to the nature of medical science. Medical science is a subject of collaborative development, and various disciplines are integrated with each other, with variances and commonalities. In the past long period of time, in order to promote the development of various medical specialties, various medical disciplines have gradually subdivided and derived many sub-specialties. The maintenance of human health, however, requires interdisciplinary collaboration since humans are organic wholes (Daiming, 2017). As a result, the idea of integrated medicine has progressively made it the development trend to improve collaboration across various specialties. Multi-disciplinary treatment and the construction of major medical centers in sample hospitals are the products of multidisciplinary collaboration. In addition to assisting clinicians in performing their responsibilities of saving lives and aiding the wounded, strong interpersonal linkages between and within clinical departments enable clinicians to fully enjoy a positive organizational climate and a feeling of organizational support. Clinicians in the sample hospital had the lowest scores for social participation and the second-lowest scores for work achievements. This is related to the daily workload and professional development characteristics of clinicians. As a university affiliated hospital, the sample hospital undertakes not only daily medical work, but also a large number of teaching and scientific research tasks. In addition, the development of clinicians themselves also needs constant learning to master more clinical skills and cutting-edge medical information. Work and study occupy most of their daily life, and it is difficult to spare time to participate in various other organizations and activities. Tertiary hospitals are places where incurable diseases are treated. In China, the effectiveness of primary care is limited (Xu et al., 2020), so patients prefer to visit doctors in tertiary hospitals as long as they are accessible and affordable. Under this circumstance, the heavy workloads can lead to job burnout among clinicians.

In this study, the three dimensions of job burnout were positively correlated with the three dimensions of DASS, and negatively correlated with the SWB, which confirms hypothesis 1–3. We also found that mental illness mediated the relationship between job burnout and poor SWB, which confirms hypothesis 4. Recognizing the mediating role of mental illness in the relationship between job burnout and poor SWB highlights the need to promote mental health promotion among clinicians through improved mental health literacy, early screening, and timely interventions. Researches have shown that non-mental health professionals have a relatively low level of mental health knowledge and literacy, which can impact their ability to provide appropriate care for patients with mental health needs and may also hinder awareness of their own mental health problems. For example, a study conducted in six general hospitals in China revealed that less than 60% of non-mental health professionals were able to correctly identify case vignettes of common mental disorders (Wu et al., 2017). Therefore, effective psychological interventions targeting clinicians need to be implemented regularly. Hospital administrators should pay more attention to the mental health problems of clinicians and improve the SWB of clinicians.

### Limitations

Several study limitations must be mentioned. On the one hand, this study was somewhat limited by its cross-sectional design, which precluded us from exploring the causal relationships between job burnout, mental illness, and SWB. On the other hand, the participants recruited from a single hospital might introduce problems related to bias and generalizability. In addition, the self-reporting of these measures may present problems related to bias and social desirability. In the future, our study could use the longitudinal study designs to track the effects of job burnout and mental illness on SWB combined with structured interviews, and conduct the intervention studies, providing a more straightforward path to how these variables interact.

## Conclusion

Job burnout and mental illness were not uncommon among clinicians in China. This study constructed a structural relationship model among job burnout, mental health and SWB. The significant association between job burnout and poor SWB, with mental illness acting as a partial mediator in this relationship. The results of this study provide a new perspective and thought on the negative effects of job burnout on SWB of clinicians. Medical administration departments and hospital administrators should prioritize the job burnout and mental health of clinicians, so as to effectively improve the SWB of clinicians.

## Data availability statement

The raw data supporting the conclusions of this article will be made available by the authors, without undue reservation.

## Ethics statement

The studies involving humans were approved by the Ethics Review Board of the Centre for Health Management and Policy Research, School of Public Health, Shandong University. The studies were conducted in accordance with the local legislation and institutional requirements. The participants provided their written informed consent to participate in this study.

## Author contributions

JW: conceptualization, critical revision, funding acquisition, supervision, writing—original draft preparation, and writing—reviewing and editing. YF: data curation, formal analysis, software, critical revision, writing—original draft preparation, and writing—reviewing and editing. DH and SZ: data curation, formal analysis, and supervision. All authors contributed to the article and approved the submitted version.
